# Spinel CoFe_2_O_4_ Nanoflakes: A Path to Enhance Energy Generation and Environmental Remediation Potential of Waste-Derived rGO

**DOI:** 10.3390/nano12213822

**Published:** 2022-10-29

**Authors:** Tamilselvi Ramasamy, Lekshmi Gopakumari Satheesh, Vaithilingam Selvaraj, Olha Bazaka, Igor Levchenko, Kateryna Bazaka, Mohandas Mandhakini

**Affiliations:** 1 Center for Nanoscience and Technology, Anna University, Chennai 600025, India; 2 International Centre for Research on Innovative Biobased Materials (ICRI-BioM)-International Research Agenda, Lodz University of Technology, 90-924 Lodz, Poland; 3 Nanotech Research Lab, Department of Chemistry, University College of Engineering Villupuram (a Constituent College of Anna University, Chennai-25), Villupuram 605103, India; 4 School of Science, College of Science, Engineering and Health, RMIT University, Melbourne, VIC 3000, Australia; 5 Plasma Sources and Application Center, National Institute of Education, Nanyang Technological University, Singapore 637616, Singapore; 6 School of Engineering, Australian National University, Canberra, ACT 2600, Australia

**Keywords:** spinel nanocomposites, photocatalysts, biomass waste derived reduced graphene oxide, specific capacitance, malachite green dye degradation

## Abstract

Carbon nanomaterials derived from agricultural waste streams present an exciting material platform that hits multiple sustainability targets by reducing waste entering landfill, and enabling clean energy and environmental remediation technologies. In this work, the energy and photocatalytic properties of reduced graphene oxide fabricated from coconut coir using a simple reduction method using ferrocene are substantially improved by introducing metallic oxides flakes. A series of cobalt ferrite rGO/CoFe_2_O_4_ nanocomposites were assembled using a simple soft bubble self-templating assembly, and their potential for clean energy applications confirmed. The transmission electron microscopy images revealed the uniform dispersion of the metal oxide on the rGO sheets. The functional group of the as synthesized metal oxide and the rGO nanocomposites, and its individual constituents, were identified through the FTIR and XPS studies, respectively. The composite materials showed higher specific capacitance then the pure materials, with rGO spinal metal oxide nanocomposites showing maximum specific capacitance of 396 F/g at 1 A/g. Furthermore, the hybrid super capacitor exhibits the excellent cyclic stability 2000 cycles with 95.6% retention. The photocatalytic properties of the synthesized rGO nanocomposites were analyzed with the help of malachite green dye. For pure metal oxide, the degradation rate was only around 65% within 120 min, while for rGO metal oxide nanocomposites, more than 80% of MG were degraded.

## 1. Introduction

The path towards “zero carbon” or “below zero” emission reduction targets is inherently linked to finding solutions for sustainable energy generation and storage, as well as mechanisms to reduce energy consumption and waste generation. Waste from agricultural industry is increasingly attracting attention for the role it may play in environmental remediation and in reducing greenhouse gas emissions as the global communities move towards a cleaner world [1,2,3]. For material synthesis, biomass waste is a valuable source of carbon and trace minerals [4,5,6], where the micro- and nanostructure of the material may give rise to unusual material architectures without the need for complex templates or multi-step processes [7,8,9,10,11,12]. With an attractive combination of properties such as good conductivity, high surface area and chemical reactivity, these multipurpose materials can underpin sustainable circular economy by enabling new efficient nanomaterial-based devices [13,14] and functional coatings [15,16,17]. In particular, the visible light driven catalytic platforms for environmental remediation [18,19], and the efficient energy storage and conversion devices [20,21] such as supercapacitors and fuel cells (e.g., direct methanol fuel cells, DMFCs) which attract tremendous interest because of the higher efficiency, low pollution emission and flexible fuel choice [22,23]. Both of these types of energy devices require electroactive materials with excellent electro-chemical activity (i.e., multiple oxidation states, low onset potential, more surface-active sites for redox reaction, etc., that affect the exchange current density) [24].

By the nature of the charge storage mechanism, supercapacitors are classified as: (i) electrical double layer capacitors (EDLC); and (ii) pseudocapacitors. For supercapacitors that store their energy by the EDLC mechanism, carbon-based materials, such as graphene, reduced graphene oxide, activated carbon, graphene oxide, carbon nanofibers and so on, are amongst the most promising cathode materials [25,26]. However, these materials are typically synthesized using input materials and techniques that are not necessarily sustainable, requiring highly pure or hazardous input materials, significant energy budgets and complex, expensive equipment [27]. In the last few decades, there has been a significant push towards replacing expensive input materials with readily available low or negative value biomass, from coffee grounds to sugarcane bagasse, rice husk, poplar wood, palm kernel shell and coconut coir [28,29,30,31]. In parallel, significant efforts have been devoted to reducing the complexity and cost of the synthesis processes so that they could actually be scaled up, while also enhancing the properties of the resultant materials.

Heteroatom doping during biomass carbonization and activation, or the creation of composites of graphene or rGO with transition metal oxides (AB_2_O_4_), e.g., CoFe_2_O_4_, can tune the electron-donor property and electrochemical behavior of the resultant carbon-based materials [32]. Amongst promising carbon/transition metal oxide composites that can be produced using in situ doping during carbonization, magnetic nanocomposites based on spinel ferrite (MFe_2_O_4_) are attractive [28,29,30,31,32,33], particularly for supercapacitor devices and applications where their removal using an external magnetic field is desirable, e.g., in medical and photocatalytic wastewater treatment [34,35,36,37,38]. Here, the interface between carbon and metallic components [39,40], as well as finely tuned morphology, high specific surface area and high pore volume facilitates contact with fluids, and in doing so prevents particle aggregation and facilitates separation of change carrier species, with the latter two issues being known limiting factors of ferrite materials when compared to e.g., modified TiO_2_ and ZnO photocatalysts [41].

Herein, we investigate the potential of cobalt ferrite CoFe_2_O_4_/rGO nanocomposites via CO_2_ bubble template method [20]. This work builds on our previous study where the energy storage and photocatalytic performance of NiFe_2_O_4_ nanoparticles were significantly improved by immobilizing them onto the rGO nanoflake-based material platform, achieving excellent specific capacitance of 599.9 F/g (at current density of 1 Ag^−1^) and retention rate of 86.5% (at 2000 cycles), as well as visible light driven photocatalytic degradation efficiency of 96.5% [20]. We also demonstrated that gas bubble templating offers significant advantages over traditional hard template- and surfactant-based methods for the synthesis of natural-resource-derived rGO materials with well-developed mesoporous nanostructure in the absence of potentially harmful or expensive chemical reducing agents and stabilizers [42,43]. Most importantly, high-quality mesoporous architectures can be attained without the need of a hard template, and associated steps of sacrificial template removal (via solvent or calcination) [44] that notably increase the complexity, length, cost and environmental footprint of the preparation processes [45].

To enhance the electrochemical properties of the resultant material, the synthesis of the metal oxide is supported on the surface of rGO sheets (through the hydrothermal method). CoFe_2_O_4_ was selected as a promising electrode material, where improved properties arise from the use of two metal elements [46,47]. The prepared CoFe_2_O_4_ materials exhibit multiple oxidation states, which enables various redox reactions [48,49,50,51,52,53]. CoFe_2_O_4_ has been previously shown to be more catalytically active when compared to NiFe_2_O_4_ nanoparticles [52], whereas other studies have shown a decreased in the cell voltage of the discharging plateaus for NiFe_2_O_4_ when compared to CoFe_2_O_4_, due to differences in the free energy formation of the oxides [53].

It should be noted that NiFe_2_O_4_ has the inverse spinel structure, with Ni^2+^ in the more active octahedral site, and CoFe_2_O_4_ has a normal spinel structure, with Co^2+^ and Fe^3+^ occupying the tetrahedral and octahedral sites, respectively. The latter also has greater coercivity and higher remanence after magnetization compared to NiFe_2_O_4_ [54]. Hence, it is worth investigating how nanocomposites comprising CoFe_2_O_4_ differ from those based on NiFe_2_O_4_ fabricated using a similar method. The various characterizations related to specific capacitance, supercapacitor cyclic stability and photo-catalytic properties of thus-synthesized spinel transition metal oxides/rGO nanocomposites were analyzed and reported.

## 2. Materials and Synthesis Methods

Reduced graphene oxide was synthesized from coconut coir waste in a single step process without using any hazardous chemicals. Coconut coir is a by-product of the coconut industry and is a rich source of cellulose, lignin, pectin, and hemicellulose. The coconut coir was washed with tap water to remove impurities, and then washed with deionized water. The cleaned material was dried, and once dried, the material was ground to powder in a mortar and mixed with ferrocene. The mixture was carbonized in a box furnace at 300 °C for 15 min [55] to produce black-colored reduced graphene oxide (rGO). The length and temperature have previously been optimized to produce high-quality material at the lowest possible energy budget, an important feasibility criterion for scale up and industrial translation. The results of the optimization process can be found in our previously published report [31]. Briefly, the carbonization temperature of 300 °C in the presence of air was chosen to avoid mass loss and decomposition of GO material, and the formation of graphite-like material [31]. Although strictly speaking, reduced graphene oxide is synthesized from graphite powder, and the carbonaceous products synthesized from coconut coir waste should be more accurately referred to as carbon sheets, we will use rGO for simplicity and to maintain consistency with published literature on the topic of waste-derived carbonaceous materials. The formed rGO was combined with various transition metal oxides like Co_3_O_4_, CoFe_2_O_4_, and a hydrothermal process was used to produce rGO/Co_3_O_4_, rGO/CoFe_2_O_4_ composites. Briefly, for rGO/CoFe_2_O_4_ material, 0.01M (0.233 g) of Co(NO_3_)_3_·6H_2_O, 0.02M (0.6464 g) of Fe(NO_3_)_2_·9H_2_O and excess amount of urea (0.1 M) were combined in a 250 mL beaker. Then, an aliquot of the ultrasonically dispersed rGO (at the concentration of 1 mg/1 mL) was added to the mixture under stirring. Cobalt (II) nitrate hexahydrate, Iron (III) nitrate nonahydrate, urea, and ammonia solution were purchased from Alfa Aesar (Lancaster, UK). Deionized water was used as solvent in all experiments. Analytical grade ethanol and acetone were used for washing purpose.

**Figure 1:**
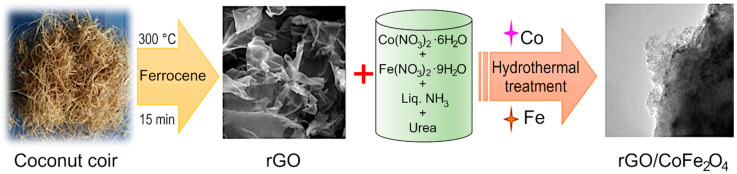
Experimental flow for the synthesis of rGO/CoFe_2_O_4_ composite.

The homogenous mixture was transferred to a stainless steel autoclave and kept for 12 h at 160 °C in a box furnace. Finally, the collected material was washed with deionized water thrice and dried overnight at 80 °C in a convection oven. Then, it was allowed to calcinate in the furnace at 300 °C for 2 h in air. Figure 1 shows the schematic representation of the synthesis procedure for the preparation of rGO/CoFe_2_O_4_. A similar protocol was used for the preparation of rGO/Co_3_O_4_ composite. For the preparation of rGO/Co_3_O_4_ composite, 0.01 M (0.233 g) of Co(NO_3_)_3_·6H_2_O excess amount of urea (0.1 M) was added, then the mixture was ultrasonically dispersed and transferred to an autoclave at 160 °C for 12 h in a box furnace. The final product was annealed at 300 °C for 2 h. From here on, the metal oxides and composites will be referred to as Co_3_O_4_, CoFe_2_O_4_, rGO/Co_3_O_4_, and rGO/CoFe_2_O_4_, respectively.

Material Characterization. The X-ray diffraction (XRD) patterns of all samples were recorded with an X-ray diffractometer (Rigaku Minflux II-c X-ray diffractometer with Cu K_α_ radiation of wavelength 0.154 nm, Tokyo, Japan) over the 2*θ* range of 10–80°.The morphologies of the as-prepared samples were characterized using scanning electron microscopy (SEM) (performed on a JEOL JSM-6700F microscope, Tokyo, Japan). Fourier transform infrared (FTIR) and RAMAN spectra were collected on a Shimadzu IRPrestige-21 infrared spectrometer (Shimadzu, Japan) over the range of 4000–500cm^−1^ and Raman Spectroscopy (AGILTRON 1(QEB1920), Agiltron Inc., Woburn, MA, USA), respectively. UV–vis adsorption spectra were recorded using a UV–vis spectrophotometer (Cary 5E, Crawley, UK) in the wavelength range of 200–1100 nm. Transmission electron microscopy (TEM) was conducted using a JEM-2100 microscope (JEOL, Tokyo, Japan) at 150 kV. X-ray photoelectron spectroscopy (XPS) was conducted on XPS HSA 15000 (Hemispherical Energy Analyzer PHOIBOS 225, SPECS GmbH, Berlin, Germany). The electrochemical studies were performed using an electrochemical analyzer (Biologic VSP, Seyssinet-Pariset, France).

Electrochemical Measurements. The electrochemical test was performed using the three-electrode system. Platinum wire and Ag/AgCl were used as the counter and reference electrodes, respectively. The working electrode was prepared with 3 mg of active material, 0.3 g of polyvinylidenefluoride (PVDF) and 0.1 mL of N-methyl pyrrolidinone (NMP) solution. The paste was coated on a nickel plate and dried for 12 h at 110 °C. The exact active mass of the material was estimated as the difference in weight between the bare and paste-coated nickel plate. Cyclic voltammogram (CV) measurements were conducted over the potential window of −0.2 to 0.6 V, with 2M of KOH solution used as the electrolyte was. The galvanostatic charge–discharge (GCD) tests were conducted between −0.2 and −0.5 V. Electrochemical impedance spectroscopy (EIS) was performed in the frequency range of 100 kHz to 0.1 Hz.

The specific capacitance *C_s_* (Fg^−1^) can be calculated based on the GCD measurement using the following equation:

*C_s_* = *IΔt*/*mΔV*, (1)

where *Δt* and *ΔV* represents the discharge time (*s*) and voltage range (*V*), respectively, *I* is the current (A), *m* is the mass of the active material (g), *C* is the capacitance of the entire supercapacitor, which is half of the *C_s_*.

## 3. Results and Discussion

**Figure 2:**
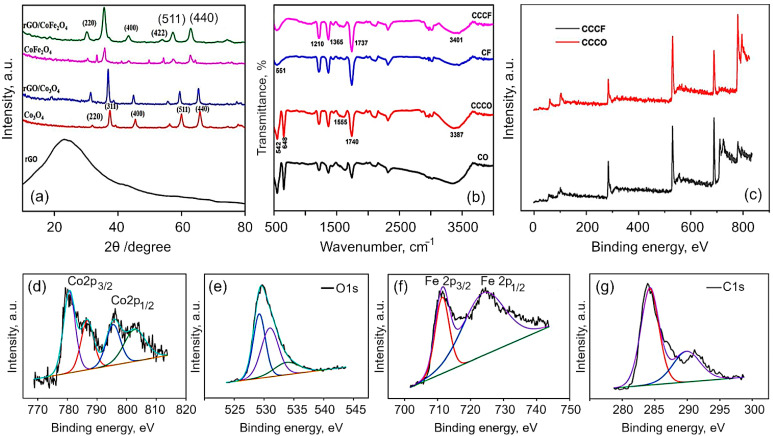
(**a**) X-ray diffraction patterns and (**b**) FTIR spectra for Co_3_O_4_, CoFe_2_O_4_, rGO/Co_3_O_4_, and rGO/CoFe_2_O_4_. The broadening of the peaks in composites is due to interactions between flakes of rGO and metal oxides. rGO is derived from coconut coir waste. (**c**) Survey spectra of rGO/Co_3_O_4_ and rGO/CoFe_2_O_4_ materials. (**d**–**g**) High-resolution XPS spectra for rGO/CoFe_2_O_4_ composite material: (**d**) Co 2p, (**e**) Fe 2p, (**f**) C 1s, and (**g**) O 1s.

Structural and chemical analysis. The XRD patterns of the obtained Co_3_O_4_, CoFe_2_O_4_, rGO/Co_3_O_4_ and rGO/CoFe_2_O_4_ composites are shown in Figure 2a. It is evident that the synthesized materials are crystalline in nature. All of the peaks in the spectra for Co_3_O_4_ can be perfectly indexed to a cubic spinel phase of Co_3_O_4_ (JCPDS no. 42-1467). Discernible peaks can be seen at 2*θ* of 31.4, 37.2, 39.1, 45.3, 56.2, 59.9 and 65.7, corresponding to (220), (311), (400), (511) and (440), respectively. In the case of CoFe_2_O_4_, the diffraction peaks were assigned in accordance with the JCPDS no. 10-0325. The main diffraction peaks of CoFe_2_O_4_ spectra appeared at 30.1, 35.7, 43.2, 54.2, 57.3, 62.6 and 74.8, corresponding to (220), (311), (400), (422), (511), (440) and (310), respectively. For the rGO/Co_3_O_4_ nanocomposites, the spectra show clear peaks at 31.3, 37.01, 38.5, 44.8, 55.7, 59.5 and 65.4. The observed shift towards the lower angle with lowering the peak intensity is due to the peak broadening, which is attributed to the presence of rGO with Co_3_O_4._ For rGO/CoFe_2_O_4_ nanocomposites, the peaks are evident at 30.07, 35.6, 43.2, 53.9, 57.3, 62.6 and 74.2, indicating peak shift towards the lower angle. Meanwhile, for both composites, the reflection peak that corresponds to (002) plain of the layered rGO has disappeared. It is speculated that the reduced graphene oxide in the rGO/Co_3_O_4_ and rGO/CoFe_2_O_4_ was fully exfoliated due to the crystal growth of Co_3_O_4_ and CoFe_2_O_4_ between the layers of the rGO sheets [31,56].

The lattice constant (a) and cell volume were calculated from XRD data, with the results shown in Table 1. The crystallite size was found to be in the range of 11.7 to 8.7nm. Briefly, the average crystallite sizes for all the synthesized nano ferrite samples were calculated using the Scherrer’s equation:

*D* = 0.9*λ*/*β* × *cos*(*θ*), (2)

where *λ* is the wavelength of X-ray radiation, *β* is full width half maximum (FWHM) in radian and *θ* is the diffraction angle.

Various peaks have been used from XRD pattern to understand the peak broadening with lattice strain. Equation (2) represents the Stokes and Wilson approach to calculate the strain induced broadening of the Bragg’s diffraction peak:

*ε* = *β*_(*hkl*)_ × *cos*(*θ*)/4*sin*(*θ*). (3)

The Willamson–Hall (WH) plots were then used to calculate the values of the grain size and strain, and the results of these calculations are tabulated in Table 1. It was observed that the crystallite sizes of Co_3_O_4_ and CoFe_2_O_4_ decreased with the changes in the lattice strain, due to which the peaks were also shifted to lower angle. It is important to mention that the crystallite size depends on the lattice strain and plays an important role in the overall properties of the nano-structure materials.

Firstly, as the crystallite size decreased, the grain boundary volume also increased with more volume defects associated with it. Secondly, with the reduction in the crystallite size, the pressure arising from the surface tension of crystallite interfaces induced stress fields that resulted in the lattice strain. From Table 1, it is apparent that in the case of rGO/Co_3_O_4_ samples, the crystallite size is 11.6 nm, and hence, much larger than the crystallite size of rGO/CoFe_2_O_4_ samples (8.7 nm). On the other hand, the surface tension-induced pressure creates a stress field in rGO/CoFe_2_O_4_ due to small crystallite size, and this in turn results in a compressive lattice strain. However, since the crystallite size is larger in rGO/Co_3_O_4_, more volume defects are associated with the grain boundaries and as a result, this ultimately exerted tensile lattice strain [57]. The presence of more defects in rGO/Co_3_O_4_ over rGO/CoFe_2_O_4_ boosted the faradaic redox reactions, and this clearly indicates that the atomic level strain effects played an important role in tuning the electrochemical properties of the developed composites, as it is discussed in the Electrochemical section below. Even though rGO/Co_3_O_4_ composites have a great number of defects, the rGO/CoFe_2_O_4_ samples showed good electrochemical and photo-catalytic performance due to the crystallize size in the rGO/CoFe_2_O_4_.

The FTIR spectra of Co_3_O_4_, rGO/Co_3_O_4_, CoFe_2_O_4_ and rGO/CoFe_2_O_4_ are shown in Figure 2b. The peaks at 1712 and 2935 cm^−1^ are assigned to the bending vibration of the C=C aromatic ring of rGO and the aliphatic C-H groups, respectively. The broad peak at 3336 cm^−1^ is the OH stretching vibration mode of OH group. In the CO spectrum, the two distinct and sharp peaks at 552.6 and 656.6 cm^−1^ correspond to the stretching vibrations of the Co^2+^ metal ions [58,59]. The absorption peaks at 1625 and 3349.6 cm^−1^ of rGO/Co_3_O_4_ composite clearly indicates the Co_3_O_4_ were successfully decorated on the surface of rGO sheets. The existence of the C=C peak in the spectra of all the rGO based samples suggests that the sp^2^ structure of the carbon atom was retained. The absorption peak at 545.4 cm^−1^ corresponds to the octahedral site Fe–O bond and tetrahedral site Co–O bond of the CoFe_2_O_4_. The spectrum of rGO/CoFe_2_O_4_ nanocomposite exhibits the characteristic peaks due to CoFe_2_O_4_ and rGO at 559 cm^−1^ and 3336 cm^−1^, respectively.

To further confirm the chemical composition of rGO/Co_3_O_4_ and rGO/CoFe_2_O_4_ composites, X-ray photoelectron spectroscopy (XPS) measurements were analyzed. The XPS spectra for rGO fabricated from coconut coir under similar conditions has previously been reported elsewhere [60,61]. Figure 2c shows the typical XPS survey spectra for the rGO/Co_3_O_4_ and rGO/CoF_2_O_4_, which reveal the presence of Co2p, Fe2p, O1s, C1s and of Co2p, O1s, C1s in rGO/CoF_2_O_4_ and rGO/Co_3_O_4_, respectively. Figure 2d–g shows the typical high-resolution spectra for Co2p, Fe2p, C1s and O1s collected for the rGO/CoFe_2_O_4_ composite. The survey spectrum of rGO/Co_3_O_4_ exhibited the peak at 282.7 eV corresponds to the C1s, 529.2 eV corresponds to the O 1s, and the peaks at 777.8 and 794eV corresponding to Co 2p_3/2_ and Co 2p_1/2_, respectively. The core level spectrum for Co exhibited two peaks corresponding to Co 2p_3/2_ and Co 2p_1/2_ at 780.6 and 795.7 eV, respectively, with two satellite peaks at 786.7 and 802.9 eV, respectively, which proved the Co^2+^ valance states [23]. The core level spectrum of Fe_2p_ shows the peaks at 711.2 and 724.5 corresponding to the Fe 2p_3/2_ and 2p_1/2_, respectively. In the C_1s_ spectrum, the peak observed at 284.2 eV corresponds to the C–C/C=C bond and the peak at 291.0 eV is attributed to the O–C=O bond. The peak at 529.1 eV can be attributed to the metal-oxygen-carbon bond, suggesting some covalent bonding with the rGO sheets. The other peaks at 531 and 534.2 eV are associated with the metal-hydroxide (M–OH) bonds [62,63]. Hence, the XPS results confirm the formation of rGO/Co_3_O_4_ and rGO/CoFe_2_O_4_ nanocomposites.

**Figure 3:**
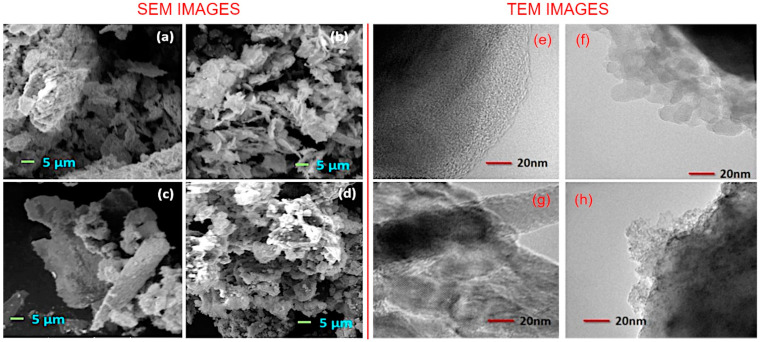
(**a**–**d**) Representative SEM images and (**e**–**h**) TEM images of (**a,e**) Co_2_O_3_, (**b**,**f**) rGO/Co_3_O_4_, (**c**,**g**) CoFe_2_O_4_, and (**d**,**h**) rGO/CoFe_2_O_4_. SEM shows flake-like morphology for Co_3_O_4_ and CoFe_2_O_4_, and incorporation of rGO and metal flakes in the composites, confirmed by TEM.

Morphological analysis. The morphology of the synthesized materials was analyzed with the scanning electron microscopy. Figure 3a–d shows the representative SEM images of the Co_3_O_4_, rGO/Co_3_O_4_, CoFe_2_O_4_, and rGO/CoFe_2_O_4_ samples. Visualization of Co_3_O_4_ and CoFe_2_O_4_ samples shows a flake-like morphology of the metal oxide particles that are homogeneous in shape, size, and how they are distributed within the mass of the material. In contrast, in the case of composites, the surfaces of rGO sheets are decorated with the metal oxide flakes. The closer observations concerning the morphology and the structure of thus-prepared materials were carried out by transmission electron microscope, which are shown in the Figure 3e–h. The incorporated nature of rGO and metal oxide nanoflakes in rGO/Co_3_O_4_ and rGO/CoFe_2_O_4_ samples is clearly visible in the TEM images. SEM image of pure rGO flakes is shown in Figure S1, Supplementary Materials (SM).

**Figure 4:**
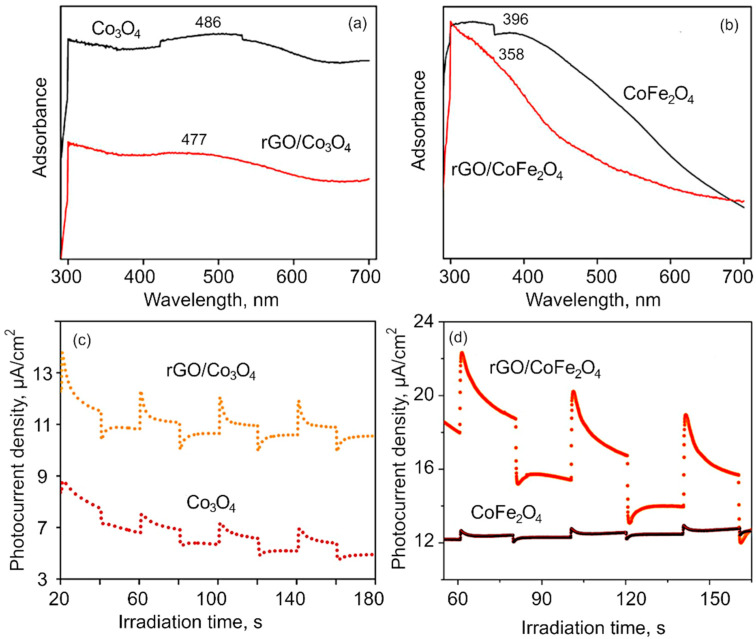
(**a**–**b**) UV–vis spectra of (**a**) Co_3_O_4_ and rGO/Co_3_O_4_ and (**b**) CoFe_2_O_4_ and rGO/CoFe_2_O_4_. The shift in spectra suggest rGO interacts chemically with the metal flakes in the composite. (**c**–**d**) Photocurrent density obtained using chronoamperometry for (**c**) Co_3_O_4_ and rGO/Co_3_O_4_, and (**d**) CoFe_2_O_4_ and rGO/CoFe_2_O_4_.

Optical characterization. The optical properties of obtained nanomaterial were investigated with UV–Vis spectroscopy. Figure 4a,b shows the UV–Vis absorbance spectra of the Co_3_O_4_, CoFe_2_O_4_, rGO/Co_3_O_4_ and rGO/CoFe_2_O_4_ nanocomposite samples. The absorption broad peak around 291.1 nm confirms the formation of reduced graphene oxide from the coconut coir waste, in alignment with previously reported results for coconut coir-derived rGO by our group [31]. The presence of the two UV–vis absorption bands at ~486 nm is ascribed to Co_3_O_4_ nanomaterial. The absorption peak corresponds to a charge transfer transition from O^2−^ to Co^2+^, i.e., band to band transition. The peak blue shift of band at 477 nm evidences that the cobalt oxide nanoflakes have been anchored onto the rGO sheets, suggesting a strong interaction between rGO and Co_3_O_4_ nanocomposites. The optical absorption spectra of the CoFe_2_O_4_ show the absorption peak at around 396 nm [64]. After incorporation with the rGO, the 396 nm peak shifted to the 331.6 nm, which evidences that rGO is not only a solid support, but also interacts chemically with the metal atoms [65,66]. Further, absorption spectroscopy plays an important role for the determination of the band gap value of the materials. It has been observed from the Figure S2 (SM) that the band gap values of the as-synthesized materials are 1.7, 1.3, 2.41, 2.48 eV for Co_3_O_4_, rGO/Co_3_O_4_, CoFe_2_O_4_, and rGO/CoFe_2_O_4_, respectively [67,68]. The spectrum for rGO flakes is shown in Figure S3 (SM).

Figure 4c,d shows the transient photocurrent measurement to investigate the charge separation and transfer efficiency through the visible light on and off region. The plots represent the photocurrent response in samples over several ON–OFF cycles. It is worth noting that despite repeated testing of ON–OFF response, the photocurrent response was found to remain similar. From the obtained results, it is evident that the photocurrent response for pure Co_3_O_4_ and CoFe_2_O_4_is very weak compared to that of their respective composites made with rGO. When rGO is integrated into the composite matrix, the improvement in the photocurrent response is attributed to an increase in the photocurrent density, illustrating that the photogenerated holes (h^+^) can be effectively separated from e^−^ in the rGO-metaloxide composite, which results in better photocatalytic performance.

**Figure 5:**
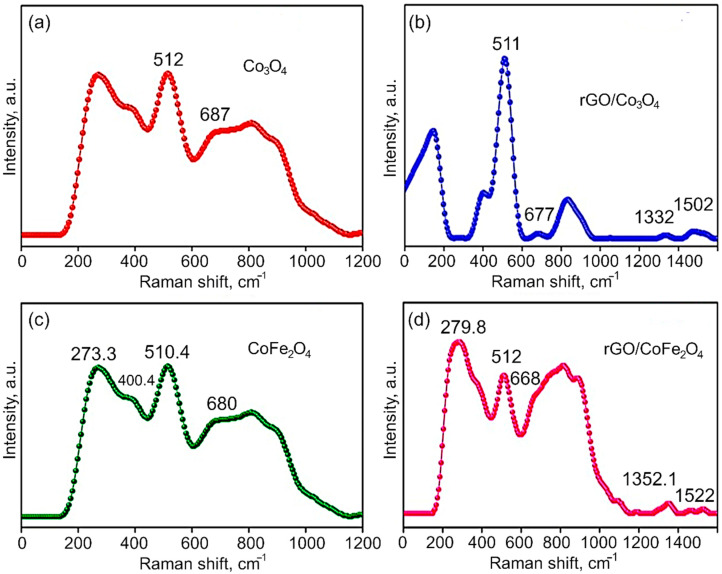
Raman spectra of (**a**) Co_3_O_4_, (**b**) rGO/Co_3_O_4_, (**c**) CoFe_2_O_4_, and (**d**) rGO/CoFe_2_O_4_.

Raman Analysis. The Raman spectra of Co_3_O_4_, CoFe_2_O_4_, rGO/Co_3_O_4_ and rGO/CoFe_2_O_4_ nanomaterials are shown in Figure 5a,b. It is known that Raman scattering is very sensitive to the microstructure of nanocrystalline materials, therefore here it is also used to further clarify the structure of the Co_3_O_4_ and CoFe_2_O_4_. In the case of Co_3_O_4_, the peaks at 512 and 677.0 cm^−1^ indicate the spinel structure of Co_3_O_4_. The Raman peaks are caused by the spinel structure’s lattice vibrations of Co^2+^ and Co^3+^ cations located in the tetrahedral and octahedral sites of the cubic lattice, respectively. From the group theory analysis, 39 normal modes of vibrations are predicted for the spinel structure; out of these modes, A_1g_ (648–680 cm^−1^), E_g_ (278–293 cm^−1^), and T_2g_ (539–565 cm^−1^ and 449–471 cm^−1^) are Raman active. From Figure 5 it is evident that the CoFe_2_O_4_ samples show Raman peaks at 273.3, 400.4, 510.4, and 680 cm^−1^, which can be assigned to the stretching vibrations of the Fe–O and Co–O bonds in the tetrahedral site of cobalt ferrite. The T_2g_ and E_g_ Raman modes are assigned to the lower frequency Raman modes, demonstrating the spinel structure. Additionally, the Raman intensity of the peaks at 1352.2 cm^−1^ (D-band) and 1522 cm^−1^ (G-band) in the composites is significantly lower than the bare metal oxides CoFe_2_O_4_ and Co_3_O_4_.

Mechanism of CoFe_2_O_4_ growth on rGO sheets. A mechanism for the assembly of rGO/Co_3_O_4_ and rGO/CoFe_2_O_4_ composites was proposed based on the outcomes of morphological, structural and chemical characterization. The assembly on metal oxide flakes on the surfaces of reduced graphene oxide sheets was generally achieved by using a hydrothermal technique with urea as a structure-directing agent [20,69,70,71]. Several investigations on the use of urea’s structure-directing property to develop metal oxides on rGO have been published, in which metal nitrates are alkalized and then hydrolyzed with NH_3_·H_2_O using the inductive effect provided by the hydrothermal process [20]. However, reports on the use of CO_2_ soft bubble template technology for this purpose are infrequent, and reports on spinel nanostructures on rGO grown in this way are not readily available. When NH_3_·H_2_O is added to a solution containing metal ions (Co^2+^, Fe^3+^), the lone pair of electrons in the NH_3_·H_2_O solution complexes with the metal ions to form [CoFe(H_2_O)_6-x_ (NH_3_)_x_]^2+^. This allows some configurational ions to be released back into the solution. When the temperature reaches 80 °C, the urea is hydrolyzed, releasing CO_2_ and NH_3_ liquid interfaces into the aqueous solution. The generated NH_3_ further drives the ionization of NH_3_·H_2_O to generate OH^−^. The generated CO_2_ bubbles are supported on rGO, and these CO_2_ bubbles on rGO act as a template for metal oxide formation. At high temperature and pressure, Fe^3+^ is reduced to Fe(OH)_3_ which converts into FeOOH. Now the precipitated Co(OH)_2_ reacts with FeOOH to form CoFe_2_O_4_ at hydrothermal conditions.

NH_3_ + H_2_O ↔ NH_3_⋅H_2_O ↔ NH^4+^ + OH^−^

Co^2+^ + xNH_3_ + (6−x) H_2_O ↔ [CoFe(H_2_O)_6−x_ (NH_3_)_x_]^2+^

CO(NH_2_)_2_ + H_2_O → 2NH_3_↑ + CO_2_↑

Co^2+^ + 2OH^−^ → Co(OH)_2_ ↓

Fe^3+^ + 3OH^−^ → Fe(OH)_3_↓

Fe(OH)_3_ → β-FeOOH + H_2_O

β-FeOOH + Co(OH)_2_ → CoFe_2_O_4_↓+ H_2_O

**Figure 6:**
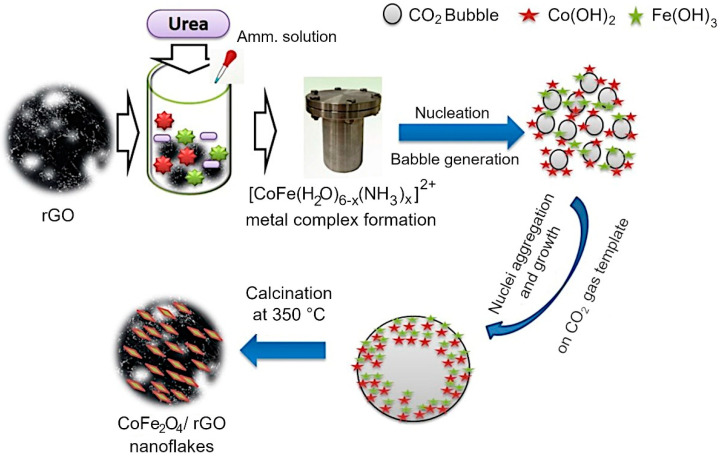
Growth mechanism of rGO/CoFe_2_O_4_ composite is enabled by the presence of urea, which decomposes upon mild heating to produce CO_2_ bubbles that act as templates for metal oxide assembly on the surfaces of rGO sheets.

Under prolonged hydrothermal conditions, the produced bubbles act as a soft template on which the Co(OH)_2_ and FeOOH aggregate to form CoFe_2_O_4_. Due to the orientation attachment, the crystal developed along a certain orientation during calcination at 350 °C for 2 h, resulting in nanoflakes. The formation of the metal oxide nanoflake is schematically illustrated in Figure 6.

**Figure 7:**
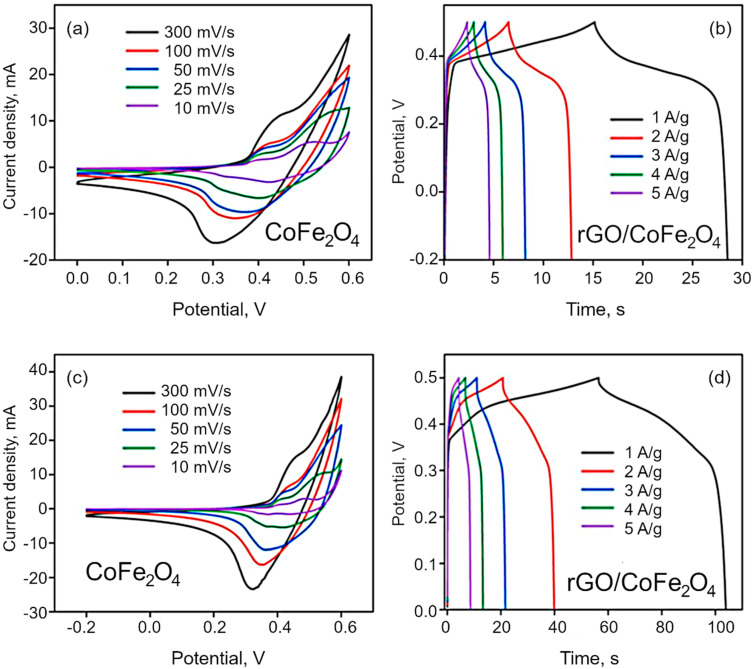
(**a**,**c**) CV curves for CoFe_2_O_4_ and rGO/CoFe_2_O_4_ at different scan rates, and (**b**,**d**) CP curves for CoFe_2_O_4_ and rGO/CoFe_2_O_4_ at different current density.

Electrochemical analysis. CV was used to characterize the electrochemical behavior of the synthesized materials. The materials show pseudo-capacitance behavior because of the cobalt and iron ions of Co_3_O_4_ and CoFe_2_O_4_, which is confirmed by the nonlinear plot of CV curves shown in Figure 7a,c. The effect of scan rate on the specific and interfacial capacitance has been studied at the rates of 10, 25, 50, 100 and 300 mV/s. The voltametric currents are increased with the scan rate, which indicates the pseudocapacitive nature of the as synthesized materials. The specific capacitance is obtained by dividing the mass of electrode dipped, whereas interfacial by dividing area of the electrode dipped in the electrolyte. The redox peak around at 0.31 V indicate the reversible redox reaction of the Co^2+^/Co^3+^ and Fe^3+^/Fe^4+^ in alkaline electrolyte. In the case of Co_3_O_4_, the redox peak is narrow than the bimetallic materials. The specific capacitance decreased with increasing scan rate because of the of inner active sites at the electrode surface not being able to maintain redox transitions as the scan rate increases. The redox reaction can be expressed by the following reactions:

Co_3_O_4_ + OH^−^ ↔ CoOOH + e^−^

CoFe_2_O_4_ + OH^−^ + H_2_O ↔ CoOOH + 2FeOOH + e^−^

CoOOH + OH^−^ ↔ FeO_2_ + H_2_O + e^−^

**Figure 8:**
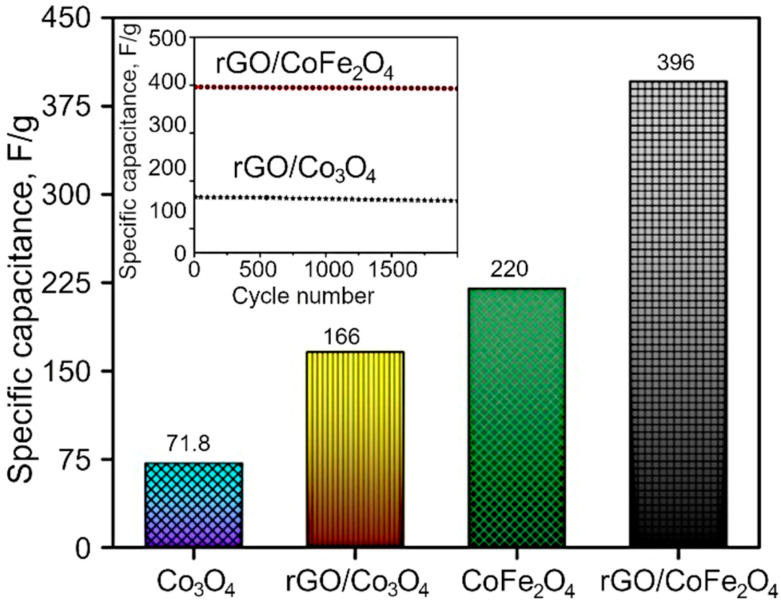
Specific capacitance of Co_3_O_4_, rGO/Co_3_O_4_, CoFe_2_O_4_ and rGO/CoFe_2_O_4_. Inset shows the cyclic stability of the rGO/Co_3_O_4_ and rGO/CoFe_2_O_4_ at 2 A/g.

The GCD curves are demonstrated in the Figure 7b,d and Figure S4, and the resulting nonlinear graphs indicate pseudocapacitive behavior for both Co_3_O_4_ and CoFe_2_O_4_. In contrast, in the case of composites, rGO/Co_3_O_4_ and rGO/CoFe_2_O_4_ display both pseudocapacitive and EDLC capacitive behavior. Here, the discharging time is higher than that for pure metal oxide materials, with a negligible IR drop due to the hybrid storage mechanism. The interactions between the electrolyte and electrode materials are also high because of the sheet-like morphology of both rGO and metal oxides. The specific capacitance of the as-synthesized materials was estimated to be 71.8, 220, 166 and 396 F/g for Co_3_O_4_, CoFe_2_O_4_, rGO/Co_3_O_4_, and rGO/CoFe_2_O_4_, respectively, as shown in Figure 8. The specific capacitance of the rGO/CoFe_2_O_4_ is higher than rGO/Co_3_O_4_ due to the number of redox reactions involved in the rGO/CoFe_2_O_4_ because of its binary metal oxide nature and rGO also offer large surface area. Furthermore, this composite is highly porous in nature, as can be seen from the microscopy images.

Figure 8 also shows the cyclic stability of the rGO/Co_3_O_4_ and rGO/CoFe_2_O_4_ at 2 A/g. The capacitance properties of the materials reduce very slightly at the high number of cycles, with excellent retention of 95.6%, which confirms excellent cyclic stability of the prepared materials. Furthermore, the enhanced electrochemical properties of the materials were analyzed with the Electrochemical Impedance Spectroscopy (EIS). Nyquist plots (Figure S5) consist of two parts, a semicircle in the high-frequency region and a nearly straight line in the low-frequency (Warburg impedance region). The radius of the semicircle represents the charge transfer resistance, while the slope of the line indicates the diffusive resistance. The small semicircle and high slope of the line confirm efficient electron transport. Among the four sample types, rGO/CoFe_2_O_4_ electrodes exhibited small charge transfer resistance, as seen in Figure 8, suggesting high electrical conductivity. These results can be due to the hybrid structure of rGO and CoFe_2_O_4_ nanoparticle integration, which could promote rapid electron and electrolyte transportation at the electrode surface. An intercept at high-frequency region with the real part (Z^−^) is attributable to a combinational resistance. The combinational resistance includes the ionic resistance of the electrolyte, the intrinsic resistance of the active material, and the contact resistance of the active material and the current collector. The combinational resistance found to be 7.6 Ω, 6.2 Ω, 5.46 Ω and 5.36 Ω for Co_3_O_4_, rGO/Co_3_O_4_, CoFe_2_O_4_ and rGO/CoFe_2_O_4_, respectively. Collectively, these results suggest that rGO/CoFe_2_O_4_ has good electrochemical properties.

Photochemical analysis. The photo-degradation potential of thus-synthesized materials was analyzed by measuring the degradation of malachite green (MG) dye in aqueous solution in the visible light photo-reactor. To assess the photocatalytic degradation process, a small amount of the photocatalyst was added to an aliquot of MG dye solution. This solution was placed in the dark with stirring to attain adsorption–desorption equilibrium for about 30 min, and then placed under visible light irradiation. During the irradiation stage, small volumes of the aqueous reaction mixture were drawn at intervals of 30 min and their UV–Vis absorption spectra collected at the corresponding wavelength of λ_max_ = 665 nm. A reduction in the magnitude of the absorption peak indicates the degradation of MG dye. The degradation percentage of the dye was calculated by using the following relation:

*E* = [1 − *A_t_*/*A*_0_] × 100, (4)

where *A_t_* is the absorbance at the time interval *t*, *A*_0_ is the absorbance at *t* = 0 min. The rate constant of photocatalytic degradation of MG for each material was obtained from the Equation (4), with the results summarized in Table 2.

**Figure 9:**
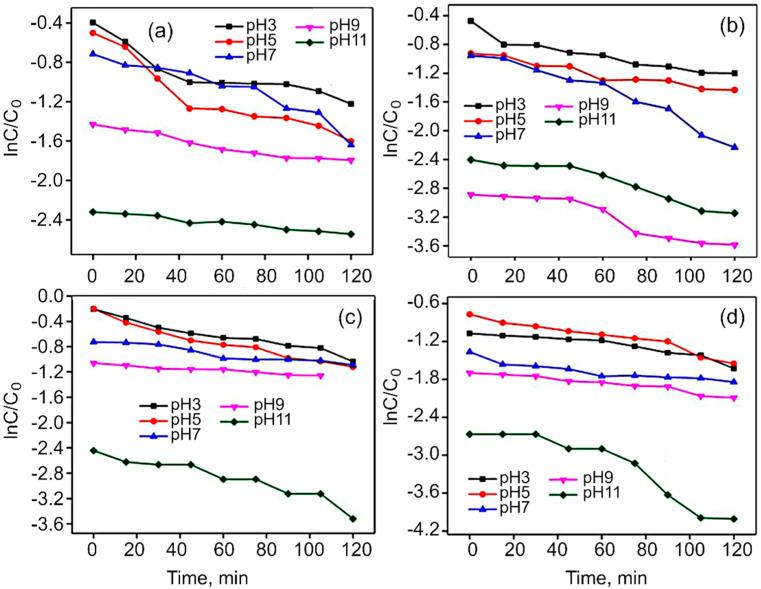
Effect of MG dye solution pH on linear kinetic curves in the presence of (**a**) Co_3_O_4_, (**b**) rGO/Co_3_O_4_, (**c**) CoFe_2_O_4_, and (**d**) rGO/CoFe_2_O_4_ catalysts.

Figure S6 shows the photocatalytic activity of Co_3_O_4_, CoFe_2_O_4_, rGO/Co_3_O_4_ and rGO/CoFe_2_O_4_ for the degradation of MG dye under the visible light irradiation. For Co_3_O_4_ and CoFe_2_O_4_, the degradation rate was only around 65% within 120 min, while for rGO/Co_3_O_4_ and rGO/CoFe_2_O_4_, more than 80% of MG were degraded. This is because rGO acts as an acceptor of the electrons generated in the metal oxide, which is suppressing the recombination of charges. The effect of pH on MG dye was studied at a fixed dye concentration and weight of the catalyst, with the solution pH varied from 3 to 11, as shown in Figure 9. The efficiency of photodegradation of MG dye linearly increases from pH 3 to 11. Malachite Green dye is a cationic dye, so in the case of the lower pH range (>7) repulsion will take place between the dye and the photocatalyst, with the breaking of the dye molecules becoming more difficult. In contrast, in the case of higher pH, the degradation takes place very easily due to the basic nature of the dye solution. This is clearly shown in the Figure S7.

The kinetic performance of the nanoflakes for the degradation of MG dye was then analyzed using the Langmuir–Hinshelwood model (Equation (5)). Figure S7 gives the chemical kinetics for the removal of MG dye that fits the pseudo-first order kinetic model ln C/C_o_ = kt, where C is the initial absorbance of MG dye at time t = 0, C_o_ is the change in absorbance of MG dye at selected intervals of degradation, and k is the first-order rate constant.

The plot of ln C/C_o_ versus irradiation time gives the rate constant value from the obtained slope. Figure S8 illustrates that the photocatalytic reaction rate constants (k) for Co_3_O_4_, CoFe_2_O_4_, rGO/Co_3_O_4_ and rGO/CoFe_2_O_4_, were 0.00436, 0.00592, 0.00513 and 0.00667 min^−1^, respectively.

These results reveal that the efficiency of the photocatalytic activity of rGO/CoFe_2_O_4_ and rGO/Co_3_O_4_ photocatalysts is increased with the incorporation of rGO sheets. The photocatalytic activity of the rGO/CoFe_2_O_4_ nanoflakes with the k value of 0.00667 min^−1^ is higher than those for the other three materials under the visible light illumination. The enhancement in the efficiency of photodegradation was observed for rGO/CoFe_2_O_4_ with the superior active surface area, which shows a greater degree of decolorizations for MG dye.

**Figure 10:**
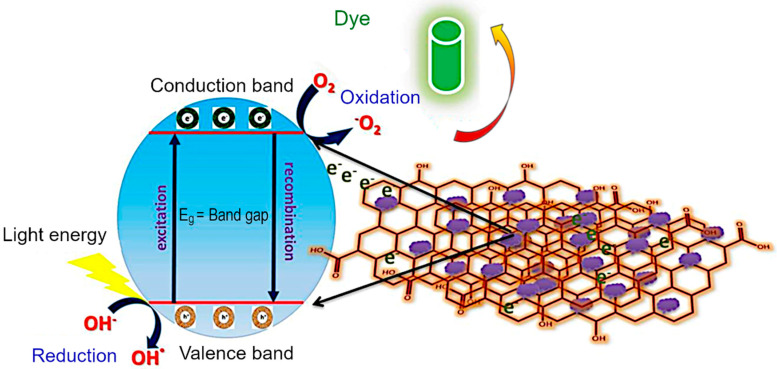
Electron transfer mechanism in rGO/CoFe_2_O_4_ catalyst during the photodegradation of MG dye.

Mechanism of dye photodegradation. The diagrammatic representation of the MG dye degradation pathway is explained in Figure 10. In a typical process, when the visible light irradiates the material, the electrons (e^−^) migrate from the conduction band (CB) to valence band (VB). Then, the holes (h^+^) are created in the valence band. The holes react with hydroxyl (OH^−^) coming from water to form •OH radicals. These •OH radicals can attack the bonds of the functional group of MG dye. In the case of pure CoFe_2_O_4_ and Co_3_O_4_, there is no external charge carrier, and so the delocalized electrons are rapidly recombined with the holes produced in the conduction band prior to being captured by OH^−^, prohibiting the further dye degradation. On the other hand, rGO/Co_3_O_4_ and rGO/CoFe_2_O_4_ nanocomposites act as charge carrier due to the presence of rGO that could effectively confine the delocalized electrons and consequently prevent the recombination of electrons and holes. On the basis of above-mentioned factors, the holes in the CB have greater possibility to react with OH− to produce OH• radicals. A much higher dye degradation performance was observed for the rGO/CoFe_2_O_4_ and rGO/Co_3_O_4_ nanocomposites. The adsorption of MG dye is supported on the surface of rGO/Co_3_O_4_ and rGO/CoFe_2_O_4_ due to π–π interaction between aromatic domain of the MG dye and rGO nanosheets. The rGO nanosheet prevents the electron hole recombination in Co_3_O_4_ and CoFe_2_O_4_ as it acts as photoelectron acceptor and as such promotes an effective photocatalytic degradation of MG molecules. The functional group can be substituted by an •OH radical forming the corresponding functional products. The sulfoxide group of dye molecule can undergo a second attack by an •OH radical producing the sulfone and causing the definitive dissociation of the two benzenic rings. Finally, an almost complete mineralization of carbon, nitrogen and sulfur hetero-atoms occurred into CO_2_, NH^4+^, NO_3_.

## 4. Conclusions

In summary, the few layered reduced graphene oxide was synthesized from coconut coir waste with a single step method. Then, the rGO/MO nanoflakes were obtained via a simple hydrothermal method, where CO_2_ bubbles formed as a result of urea decomposition played an important role of a template during metal oxide flake synthesis in the surface of rGO. The as-prepared Co_3_O_4_ and CoFe_2_O_4_ yielded specific capacitances of 71.8 and 220 F/g at 1 A/g. The obtained rGO was employed as advanced support to grow CoFe_2_O_4_ and Co_3_O_4_ for achieving ideal capacitive performances. The optimal rGO/CoFe_2_O_4_ composite delivered 396 F/g at 1 A/g, and demonstrated excellent cycling performance, with 94.5% of the capacitance retained after 2000 cycles. The few layered rGO nanosheets offer high surface area leading to high electrical conductivity and improved mechanical and cyclic stability of the composites. Therefore, the ultrahigh specific capacitance occurs due to the effective utilization of active material CoFe_2_O_4_ on the conductive rGO sheets. The photocatalytic results show that rGO/CoFe_2_O_4_ and rGO/Co_3_O_4_ are excellent photocatalysts for the visible light photodegradation of MG dyes compared to bare Co_3_O_4_ and CoFe_2_O_4_ catalysts. This work is of particular interest to those working in the area of valorization of biomass waste, who look to enhance the properties of biomass-derived advanced materials and expand their potential applications in the area of green energy and environmental remediation, hence fully embracing the concept of “Waste to Treasure”.

## Figures and Tables

**Table 1:** Physical parameters of Co_3_O_4_, CoFe_2_O_4_, rGO/Co_3_O_4_, and rGO/CoFe_2_O_4_ samples calculated from XRD data.

Sample Type	Lattice Parameter Å	Scherrer Crystallite Size D, nm	WH Crystallite Size D, nm	Strain ε, %
Co_3_O_4_	8.01	1.5854	4.7 ± 0.009	3
rGO/Co_3_O_4_	8.07	1.115	11.6 ± 0.002	2
CoFe_2_O_4_	5.83	1.601	9.2 ± 0.002	0
rGO/CoFe_2_O_4_	5.85	1.315	8.7 ± 0.002	0.4

**Table 2:** Malachite Green dye visible light photodegradation efficiency and rate constant values for Co_3_O_4_, rGO/Co_3_O_4_, CoFe_2_O_4_, and rGO/CoFe_2_O_4_.

Catalysts	Rate Constant (k), min^−1^	Degradation Efficiency, %
Co_3_O_4_	0.00436	66.2
rGO/Co_3_O_4_	0.00592	68.1
CoFe_2_O_4_	0.00513	72.7
rGO/CoFe_2_O_4_	0.00667	80.8

## Data Availability

The data presented in this study are available on request from the corresponding author.

## Supplementary Materials

The following supporting information can be downloaded at: https://www.mdpi.com/article/10.3390/nano12213822/s1, Figure S1: SEM image of pure rGO flakes; Figure S2: Tauc plots for (a) Co_2_O_3_ and rGO/Co_2_O_3_, and (b) CoFe_2_O_4_ and rGO/CoFe_2_O_4_. Absorption data obtained using UV-Vis spectroscopy. E_g_ estimated as the intercept (y=0) between the linear fit to plot generated using the Tauc equation αhν= B (hν − E_g_)^n^, where α is the optical absorbance, ν is the frequency of light, and n and B correspond to the type of transition and the length of localized state tails respectively; Figure S3: Spectrum for rGO flakes; Figure S4: (a, c) Representative CV curves for Co_3_O_4_ and rGO/Co_3_O_4_ samples at different scan rates. (b, d) Representative CP curves for Co_3_O_4_ and rGO/Co_3_O_4_ samples at different current density; Figure S5: Nyquist plots; Figure S6: Degradation of MG dye solution under visible light irradiation as a function of solution pH; Figure S7: Changes in the visible spectra of MG at different irradiation time; Figure S8: Photocatalytic activities (PDF).
