# Supplementary material for: Spinel CoFe2O4 Nanoflakes: A Path to Enhance Energy Generation and Environmental Remediation Potential of Waste-Derived rGO
Source: Nanomaterials (Basel). 2022 Oct 29;12(21):3822. doi: 10.3390/nano12213822 (PMC9657719; doi:10.3390/nano12213822)
Supplement: Supplementary file 1 [file nanomaterials-12-03822-s001.zip › nanomaterials-1913967-supplementary.pdf]

Supplementary information

Spinel $CoFe_2O_4$ nanoflakes: A path to enhance energy generation and environmental remediation potential of waste-derived rGO

R. Tamilselvi[1], G. S. Lekshmi[1], V. Selvaraj[2], O. Bazaka[3], I. Levchenko[4], K. Bazaka[5], M. Mandhakini[1,*]

[1]Center for Nanoscience and Technology, Anna University, Chennai- 600 025, India

[2]Nanotech Research Lab, Department of Chemistry, University College of Engineering Villupuram (A Constituent College of Anna University, Chennai-25), Villupuram-605 103, Tamil Nadu, India

[3]School of Science, College of Science, Engineering and Health, RMIT University, Melbourne, VIC 3000, Australia

[4]Plasma Sources and Application Center, NIE, Nanyang Technological University, Singapore

[5]School of Engineering, Australian National University, Canberra ACT 2600

*Corresponding author (mandhakini7@gmail.com)

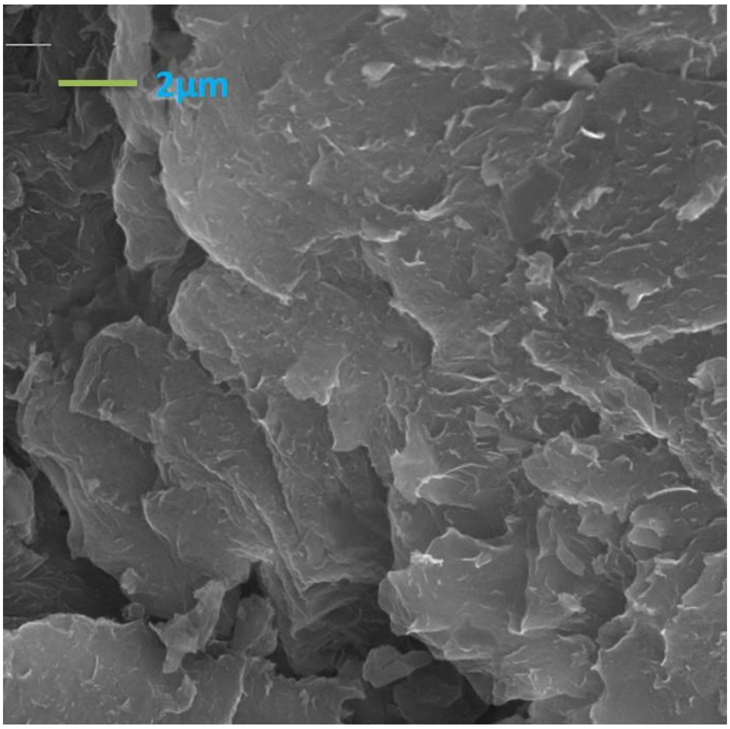

Figure S1. SEM image of pure rGO flakes.

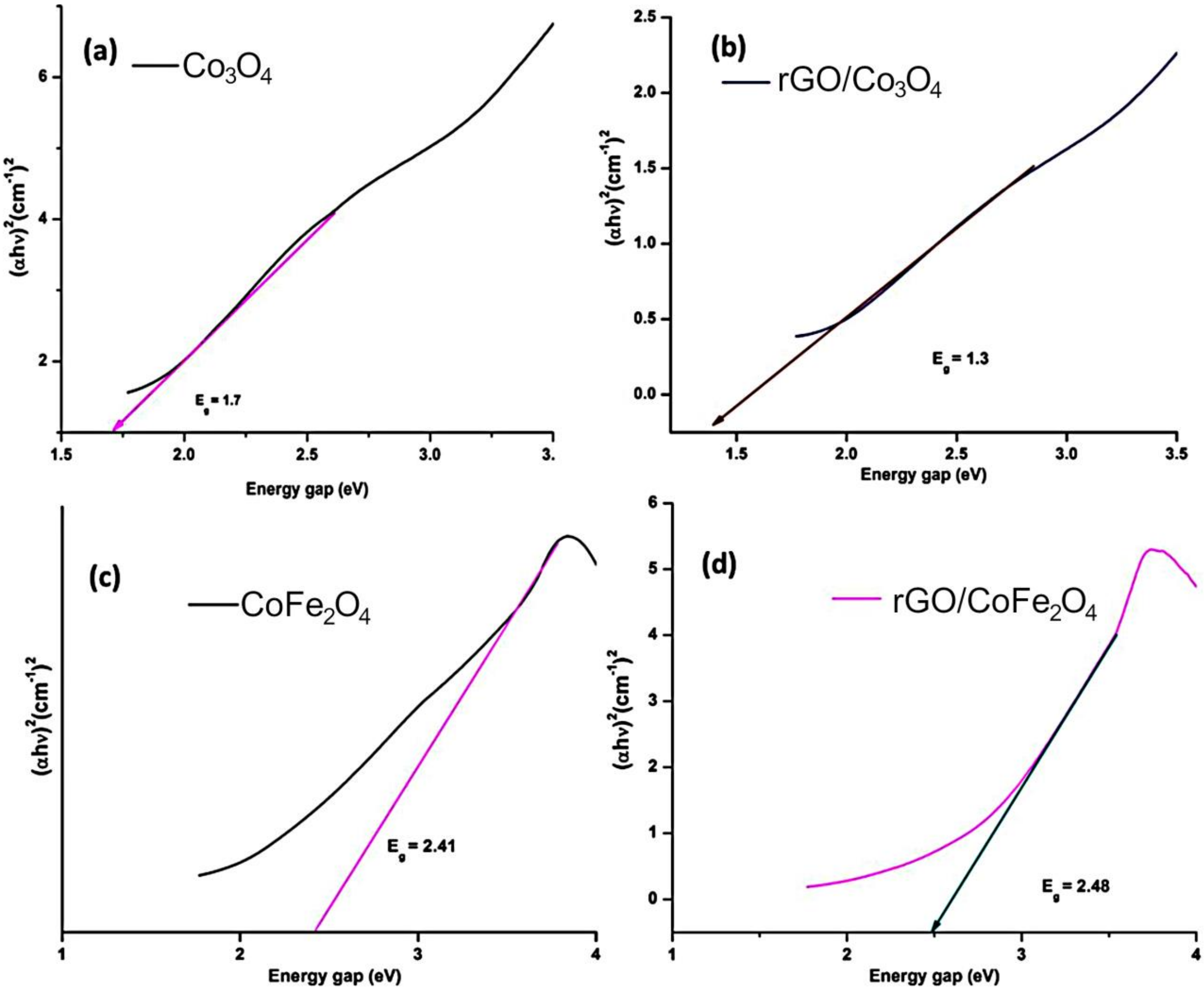

Figure S2. Tauc plots for (**a**) Co_2O_3 and rGO/Co_2O_3, and (**b**) $CoFe_2O_4$ and rGO/$CoFe_2O_4$. Absorption data obtained using UV-Vis spectroscopy. E_g estimated as the intercept (y=0) between the linear fit to plot generated using the Tauc equation $\alpha h\nu = B\,(h\nu - E_g)^n$, where α is the optical absorbance, ν is the frequency of light, and n and B correspond to the type of transition and the length of localized state tails respectively.

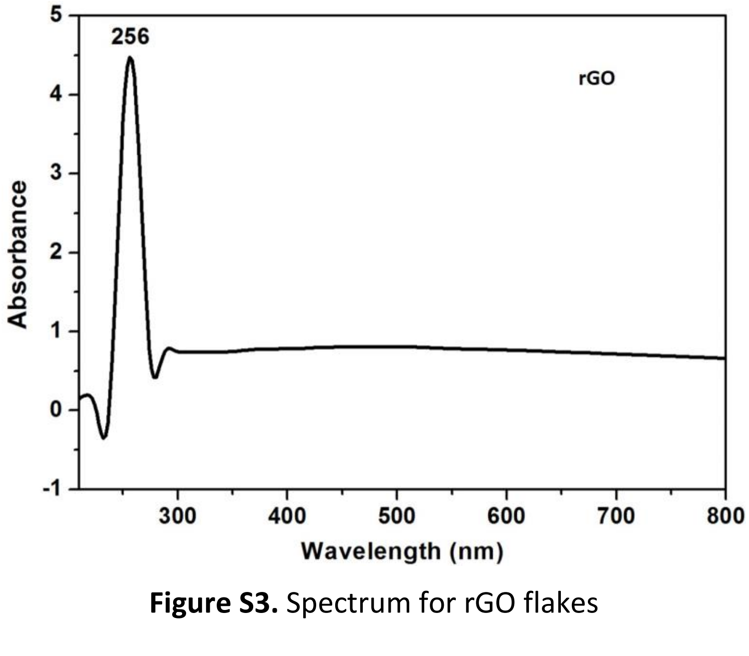

Figure S3. Spectrum for rGO flakes

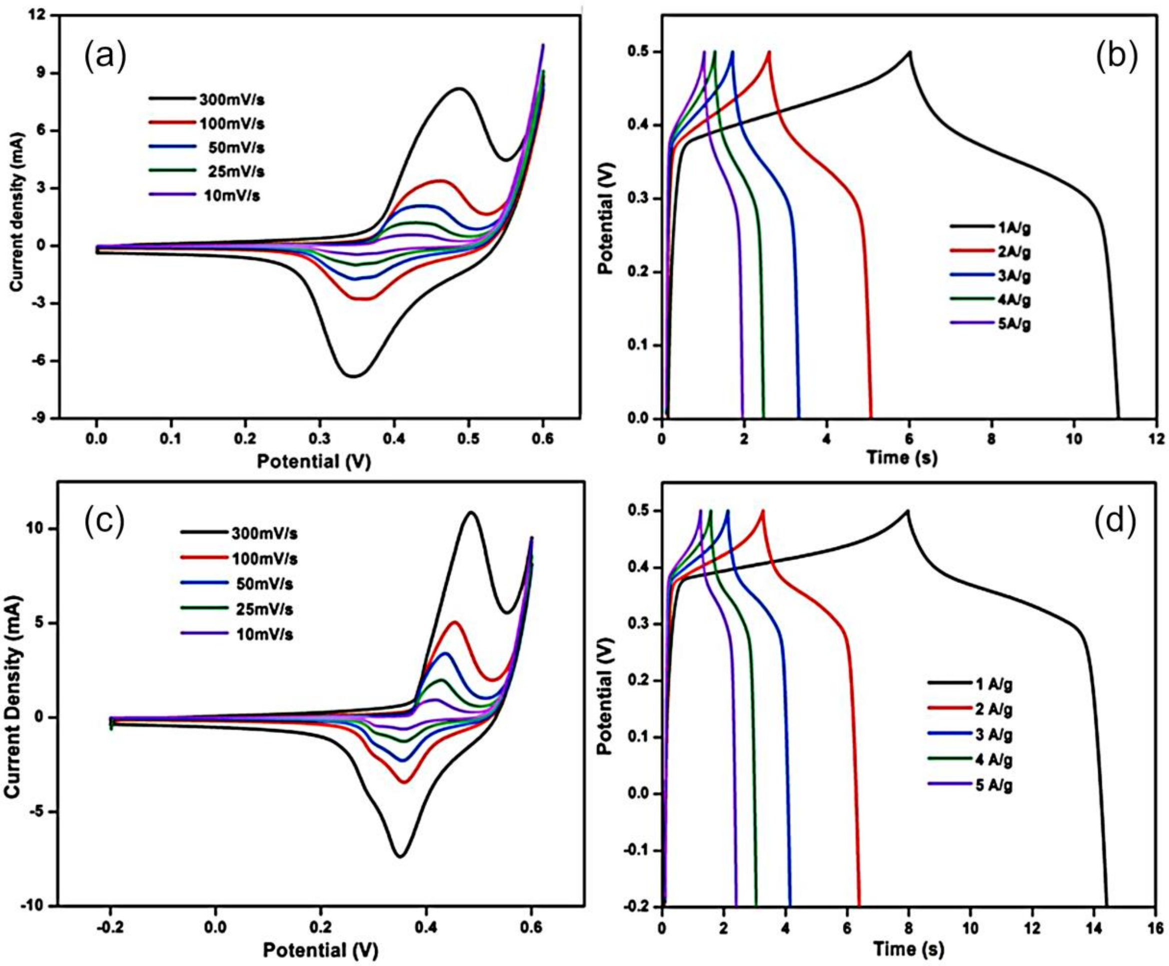

Figure S4. (a, c) Representative CV curves for Co_3O_4 and rGO/Co_3O_4 samples at different scan rates. **(b, d)** Representative CP curves for Co_3O_4 and rGO/Co_3O_4 samples at different current density.

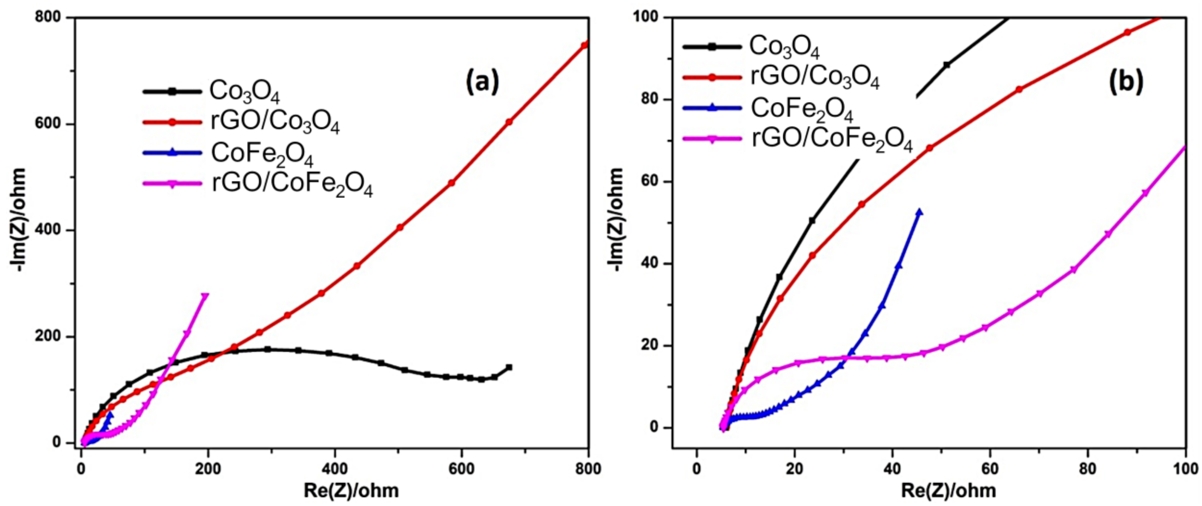

Figure S5. Nyquist plots for rGO/$CoFe_2O_4$, $CoFe_2O_4$, rGO/Co_3O_4, and Co_3O_4, (d) Expanded view.

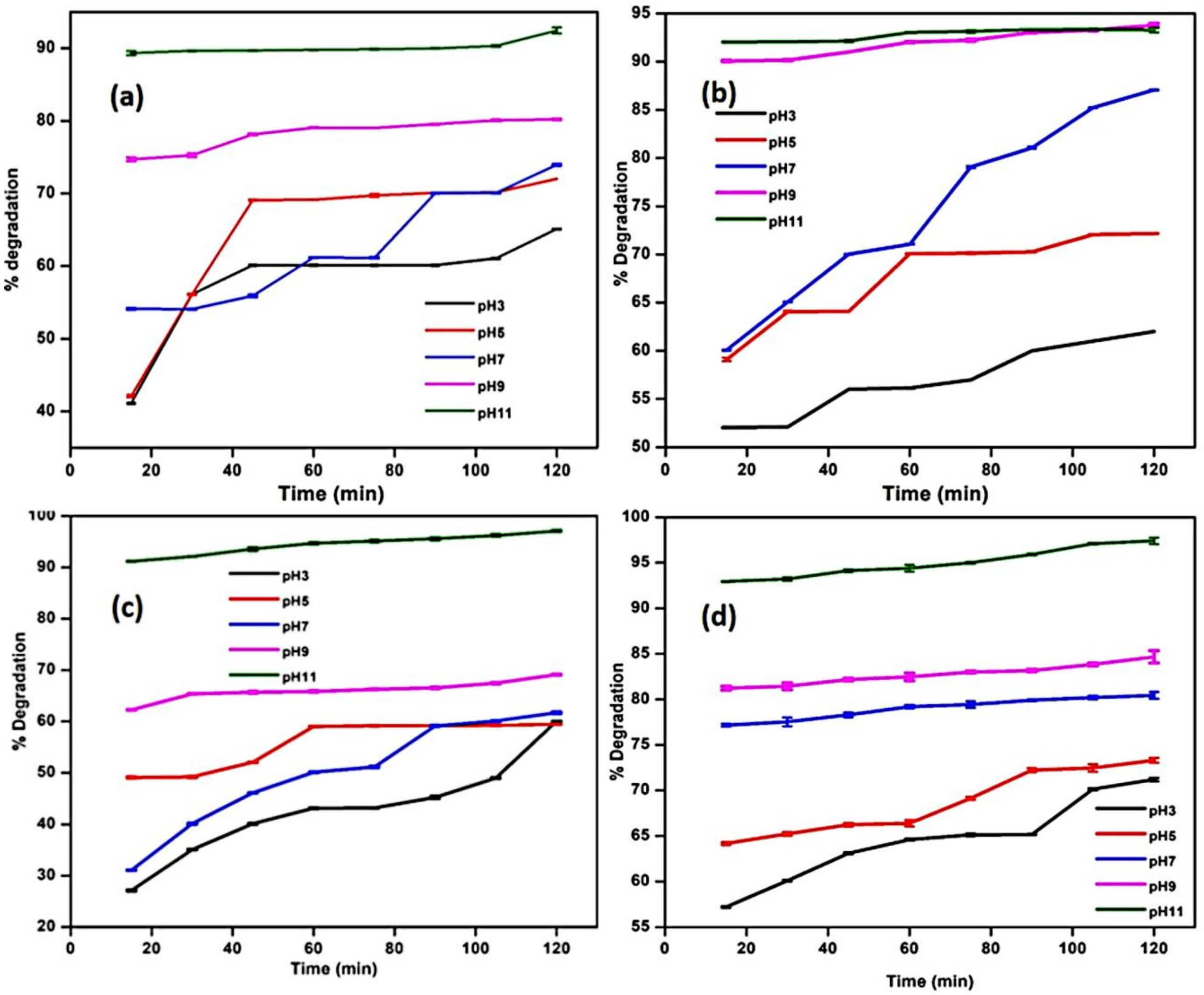

Figure S6. Degradation of MG dye solution by Co_3O_4, rGO/Co_3O_4, $CoFe_2O_4$, and rGO/$CoFe_2O_4$ catalysts under visible light irradiation as a function of solution pH.

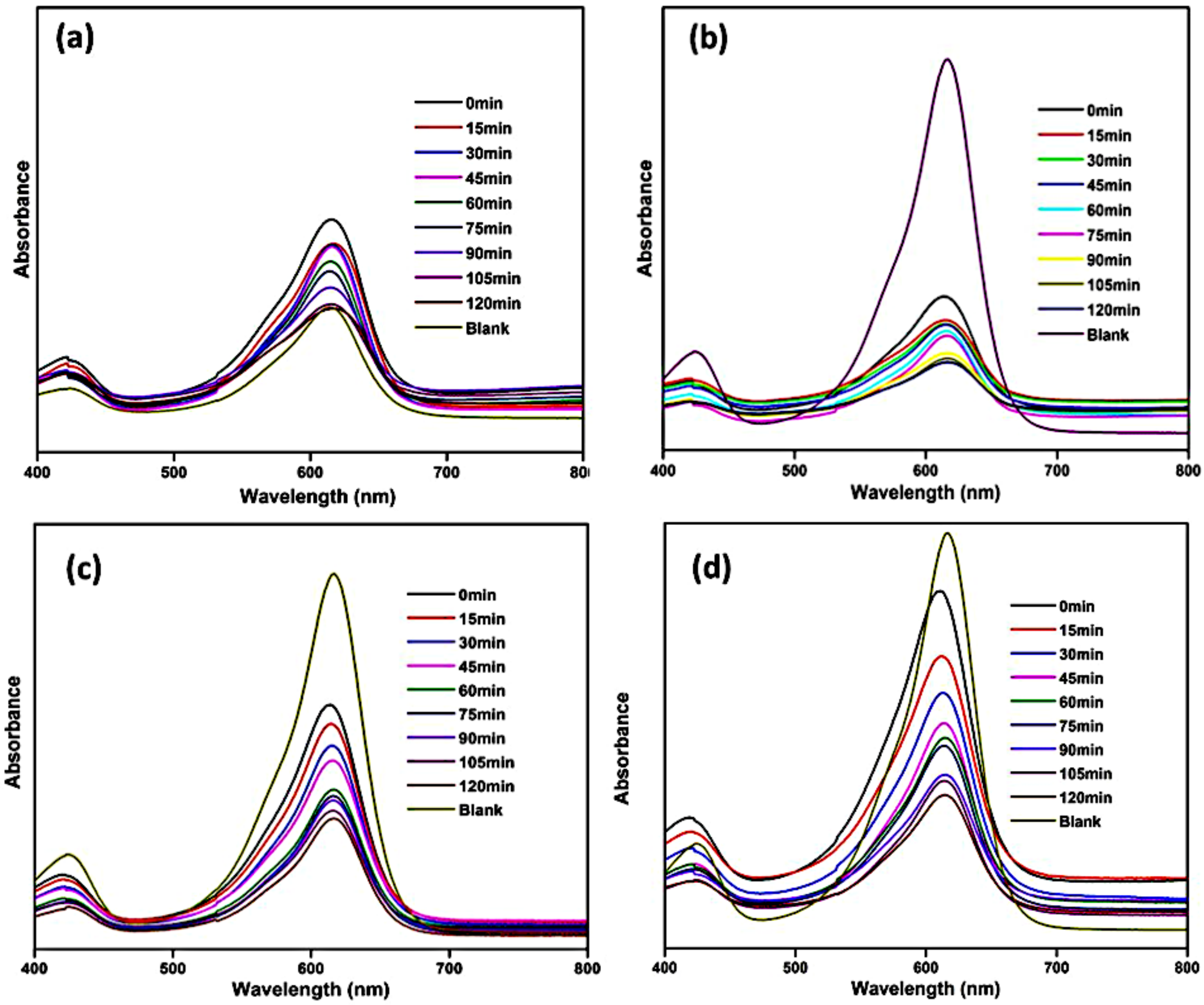

Figure S7. (a-d) Changes in the visible spectra of MG at different irradiation time in the presence of Co_3O_4, rGO/Co_3O_4, $CoFe_2O_4$, and rGO/$CoFe_2O_4$.

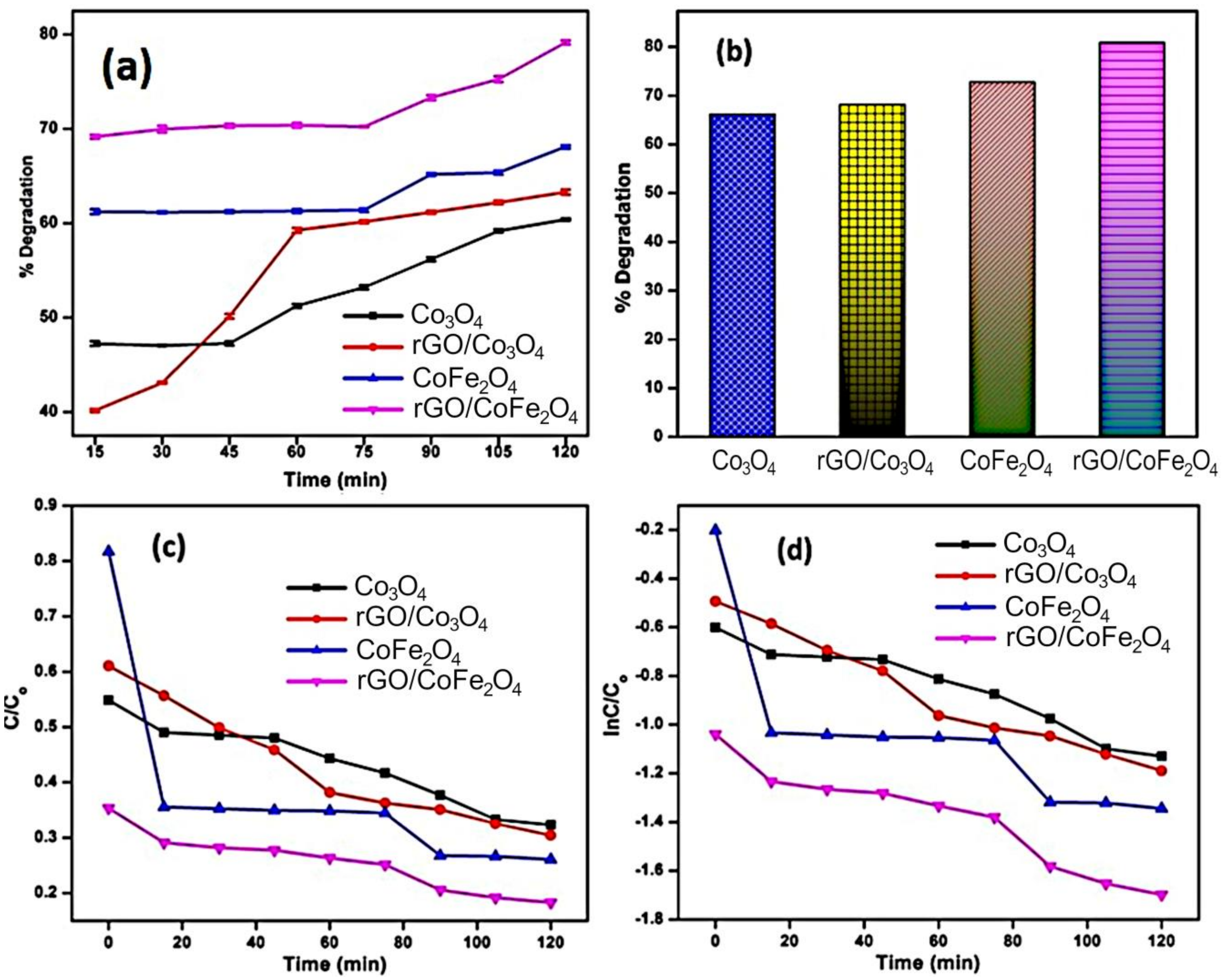

Figure S8. Photocatalytic activity of Co_3O_4, rGO/Co_3O_4, $CoFe_2O_4$, and rGO/$CoFe_2O_4$ catalysts as a function of degradation time.
